# Pharmacological inhibition of RORγt suppresses the Th17 pathway and alleviates arthritis *in vivo*

**DOI:** 10.1371/journal.pone.0188391

**Published:** 2017-11-20

**Authors:** Ulf Guendisch, Jessica Weiss, Florence Ecoeur, Julia Christina Riker, Klemens Kaupmann, Joerg Kallen, Samuel Hintermann, David Orain, Janet Dawson, Andreas Billich, Christine Guntermann

**Affiliations:** 1 Autoimmunity, Transplantation and Inflammation, Novartis Institutes for BioMedical Research, Basel, Switzerland; 2 Chemical Biology & Therapeutics, Novartis Institutes for BioMedical Research, Basel, Switzerland; 3 Global Discovery Chemistry, Novartis Institutes for BioMedical Research, Basel, Switzerland; University of South Florida St Petersburg, UNITED STATES

## Abstract

Retinoic acid receptor-related-orphan-receptor-C (RORγt) is the key transcription factor that is driving the differentiation of IL-17 producing T-helper 17 (Th17) cells that are implicated in the pathology of various autoimmune and inflammatory diseases. Based on the importance of RORγt in promoting Th17-driven pathology, there is considerable interest to develop low-molecular-weight compounds with the aim of inhibiting the transcriptional activity of this nuclear hormone receptor. In this article, we describe the *in vitro* and *in vivo* pharmacology of a potent and selective small-molecular-weight RORγt inverse agonist. The compound binds to the ligand binding domain (LBD) of RORγt leading to displacement of a co-activator peptide. We show for the first time that a RORγt inverse agonist down-regulates permissive histone H3 acetylation and methylation at the *IL17A* and *IL23R* promoter regions, thereby providing insight into the transcriptional inhibition of RORγt-dependent genes. Consistent with this, the compound effectively reduced IL-17A production by polarized human T-cells and γδT-cells and attenuated transcription of RORγt target genes. The inhibitor showed good *in vivo* efficacy in an antigen-induced arthritis model in rats and reduced the frequencies of IL-17A producing cells in *ex vivo* recall assays. In summary, we demonstrate that inhibiting RORγt by a low-molecular-weight inhibitor results in efficient and selective blockade of the pro-inflammatory Th17/IL-17A pathway making it an attractive target for Th17-mediated disorders.

## Introduction

**Fig 1:**
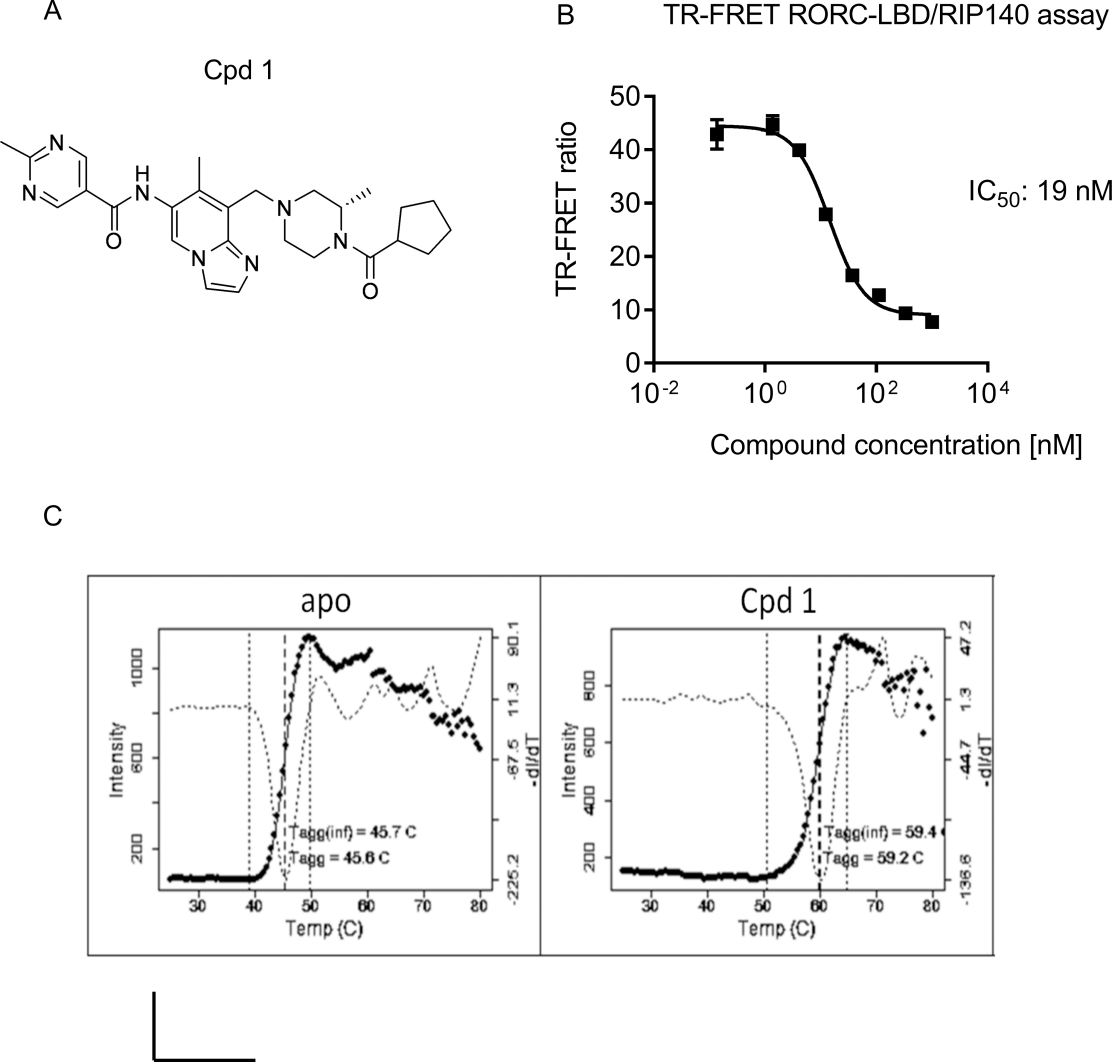
Cpd 1 is a potent RORγt inhibitor and binds to the ligand binding pocket of RORγt. (A) Chemical structure of low-molecular-weight cpd 1. (B) TR-FRET assay measuring RIP140 co-factor displacement from the human RORγt-LBD by cpd 1. Representative concentration-dependent curve from ten independent experiments with duplicate readings are shown. Inhibitory concentration to achieve 50% of the signal (IC_50_) is indicated in the graph. (C) Results of thermal stability studies by DSLS. Solid lines show the scattered light intensity as a function of temperature. The dotted line is the negative of the 1^st^ derivative of this function, with a minimum at the inflection point, indicating the value of Tagg(inf), which characterizes the midpoint of the transition. A comparison with apo shows that cpd 1 binds with high affinity to the RORγt-LBD leading to ΔTagg(inf) of 13.7 ^o^C. All experiments were performed independently for at least three times with similar results.

CD4^+^ Th17 cells are characterized by the production of effector cytokines IL-17A, IL-17F, IL-22, GM-CSF, and, to a lesser extent, tumor necrosis factor (TNF) and IL-6 [1]. In addition to promoting autoimmune inflammation, Th17 cells are critical for host immunity against fungi and extracellular bacteria [2, 3]. Differentiation and functionality of Th17 cells require the expression of the `master`transcription factor, retinoic acid receptor-related orphan receptor gamma t (RORγt), the T-cell-specific RORγ isoform, which is induced upon stimulation of naïve CD4^+^ T-cells by TGF-β and IL-6 [4, 5]. RORγt regulates the expression of the Th17 signature cytokines IL-17A, IL-17F, IL-22 as well as IL-23 receptor, CCL20 and CCR6 [4, 6, 7]. In addition to Th17 cells, expression of RORγt and its target cytokines have been reported in other cell types, such as CD8^+^Tc17 cells, invariant natural killer T-cells, ILC3 and γδ T-cells [8, 9]. There is a growing appreciation that both Th17 and RORγt-expressing innate-like lymphoid cells are important players in the pathogenesis of several human autoimmune diseases [2, 9]. Antagonizing this pro-inflammatory pathway by antibodies directed against the involved cytokines such as IL-17A and IL-23 or their receptors have demonstrated clinical efficacy in psoriasis, psoriatic arthritis, autoimmune uveitis, ankylosing spondylitis and Crohn`s disease [10–13]. RORγt has emerged as a highly attractive drug target in Th17 cell-mediated diseases due to its pivotal role in the IL-17/IL-23 axis and because its activity can be modulated by small-molecular-weight inverse agonists binding to the RORγt ligand-binding pocket. In mouse models, genetic deficiency of RORγt results in protection of experimental autoimmune encephalomyelitis (EAE), T-cell-transfer-mediated colitis and leads to profound defects in Th17 differentiation [4, 14]. Several small-molecular-weight inhibitors targeting RORγt have been discovered and were shown to suppress the Th17/IL-17 pathway as well as alleviating pro-inflammatory diseases in various mouse models such as EAE and intestinal and skin inflammation [15–20]. In a previous communication, we reported identification of a novel imidazopyridine series of potent and selective RORγt inverse agonists by an extensive structure-based optimization campaign [21]. In this report, we describe the in-depth characterization of cpd 1 (Fig 1A, designated **10** in ref. 21), the lead example of this series, focusing on RORγt-dependent responses *in vitro* and *in vivo*.

We demonstrated that the inhibitor bound to the RORγt LBD and efficiently displaced the co-activator peptide derived from receptor interacting protein 140 (RIP140), in a Foerster resonance energy transfer assay. The ability of the compound to disrupt the interaction with the co-activator peptide correlated well with the inhibition of Th17 and Tc17 cell differentiation, as well as with the suppression of acute IL-17A secretion from human γδ T-cells. Other cytokines that were down-regulated in Th17 cells after pharmacological inhibition of RORγt included IL-17F and IL-22. Furthermore, cpd 1 attenuated expression of genes such as *IL26*, *IL23R* and *CCR6* in primary human Th17 cells, which are known to be regulated by RORγt. At a molecular level, the RORγt inhibitor interfered with the epigenetic regulation of the *IL17A* and *IL23R* gene transcription by suppressing histone H3 acetylation (H3Ac) and trimethylation of lysine4 on histone H3 (H3K4me3) at their promoter regions. The compound did not affect the ability of RORγt to interact with its cognate DNA binding sites. The inverse agonist was selective for RORγt and showed no inhibitory activity against the closely related nuclear hormone receptors RORα or RORβ. In addition, cpd 1 had favorable physicochemical properties and adequate oral bioavailability and showed efficacy in a T-cell mediated *in vivo* mechanistic model. The RORγt inhibitor was able to attenuate the knee swelling response in an antigen-induced arthritis (AiA) model performed in rats and inhibited IL-17A cytokine production in *ex vivo* recall assays. These results illustrate that pharmacological inhibition of RORγt by a low-molecular-weight antagonist can be a tractable approach for the treatment of IL-17A-dependent autoimmune and inflammatory diseases.

## Materials and methods

### Human and animal study approval

Blood from healthy volunteers was provided under informed consent and collected through the Novartis Tissue Donor Program (TRI0128) in accordance with the Swiss Human Research Act and approval of the responsible ethic committee (Ethikkommission Nordwest- und Zentralschweiz number: 329/13). Buffy coats from healthy volunteers were provided under informed consent and collected through the InterRegionale Blutspende of the Swiss Red Cross. Animal housing and studies were performed in accordance with the Swiss Animal Welfare law, issued by the Kantonal Veterinary Office of Basel Stadt, Switzerland (licence number BS-1438).

### Animals

Female or male Lewis rats (approximately 120 to 150 g at start of dosing) were purchased from Charles River Laboratories. Animals were housed in enriched environments with an approximate 12-hr light/dark cycle, with food and water provided *ad libitum*.

### Synthetic chemistry

Cpd 1 [(*S*)-N-(8-((4-(cyclopentanecarbonyl)-3-methylpiperazin-1-yl)methyl)-7-methylimidazo[1,2-a]pyridin-6-yl)-2-methylpyrimidine-5-carboxamide] was synthesized as described in [21].

### Differential static light scattering analysis

The thermal stability of RORγt-ligand complexes was measured by differential static light scattering (DSLS) using StarGazer (Harbinger Biotechnology and Engineering Corporation, Markham, Canada). This method assesses protein stability by monitoring protein aggregation during thermal protein denaturation.

The LBD of human RORγt comprising the amino acids 264–518 was incubated with cpd 1 for 20 min in a 25 μl volume in a clear-bottom 384-well plate (Nunc 242764). The final condition in the well was 6 μM RORγt-LBD protein, 60 μM compounds, 4% DMSO, 20 mM Hepes pH 7.5, 100 mM NaCl, 1 mM Tris(2-carboxyethyl)phosphine (TCEP). Wells were covered with 25 μl mineral oil (Sigma) to prevent evaporation, then the plate was heated from 25 to 80°C at 1°C/min. Protein aggregation was monitored by tracking the change in scattered light that was detected by a CCD camera. Images of the plate were taken every 0.5°C. The pixel intensities in a preselected region of each well were integrated using image analysis software to generate a value representative of the total amount of scattered light in that region. These intensities were then plotted against temperature for each sample well and fitted to obtain the aggregation temperature (Tagg) as described elsewhere [22].

### Time resolved fluorescence Foerster resonance transfer assay (TR-FRET)

The ability of the compound to disrupt the interaction between the human RORγt-LBD (264–518, produced at Novartis) with a biotinylated RIP140 derived co-activator peptide (biotinyl-NH-Ahx-NSHQKVTLLQLLLGHKNEEN-CONH2, Thermo Scientific) was assessed by TR-FRET as described in Refs [21, 23]. Briefly, compounds were incubated for 1 hr in an assay mixture containing 5 nM His_6_-tagged RORγt-LBD, 90 nM biotinylated RIP140 co-activator peptide, 0.45 nM Cy5-labelled streptavidin (GE Healthcare), 1.5 nM Europium-labelled anti-His_6_ antibody (PerkinElmer), followed by measuring the emissions at 615 nm and 665 nm. Concentration-response curves were plotted and IC_50_ values were calculated using the GraphPad Prism software package.

### Lentiviral transduction of HUT78 T-cell line

Human full-length eGFP-RORγt or eGFP-empty constructs were cloned into lentiviral vectors [24], kindly provided by Prof. L. Naldini (San Raffaele Telethon Institute for Gene Therapy and San Raffaele Scientific Institute, Milano, Italy). Viral stocks were produced by Lipofectamine-mediated transfection of HEK293T cells. The human HUT78 T-cell line (purchased from the American Type Tissue Collection) was transduced with concentrated lentiviral vector stocks at a multiplicity of infection of 5 in the presence of polybrene (8 μg/ml). Plates were centrifuged for 1 hr at 2000 rpm, at room temperature followed by incubation of cells at 37°C for 48 hrs before the supernatants were replaced with fresh medium. Single cell clones expressing high levels of eGFP-RORγt were obtained by FACS sorting and were subjected to limiting dilution. For compound testing, HUT78 cells stably expressing RORγt (5 x 10^4^ / well) were stimulated with PMA (4 ng/ml, Sigma) plus CD3 mAb (2.5 μg/ml, clone OKT-3, Bioxell) and incubated in X-vivo 15 medium containing 10% FCS with diluted compounds. After 48 hrs of incubation at 37°C, the supernatants were collected to quantify IL-17A by ELISA according to the manufacturer`s specifications (eBioscience).

### RORα and RORβ GAL4 reporter gene assays

To assess selectivity against other ROR family members, human RORα or RORβ-LBD/ GAL4-DNA binding domain (DBD) reporter gene assays were performed as previously described [23]. Briefly, Jurkat cells stably expressing the pGL4.35 plasmid (Promega) containing nine GAL4 upstream activator sequences, which induce the transcription of the luciferase reporter gene were transfected with RORα or RORβ-LBD/GAL4-DBD constructs. Cells were incubated with cpd 1 and after 24 hrs of incubation, cells were lysed and luciferase activity was measured by an EnVision Multilabel Plate Reader (Perkin Elmer).

### Human Th17 and Tc17 cell isolation and T-cell differentiation

PBMCs were obtained from human buffy coats by density gradient centrifugation using Ficoll-Paque. Total CD4^+^ or CD8^+^ T-cells were purified by immunomagnetic isolation using a CD4^+^ or CD8^+^ T-Cell Enrichment Kit according to the manufacturer’s instructions (Stem Cell). Memory or naïve CD4^+^ T-cells were isolated using a memory or naïve CD4^+^ T-cell Enrichment Kit (Stem Cell), respectively. For T-cell stimulation experiments, cells were treated as described [23]. Briefly, cells were incubated in microtiter plates that had been pre-coated with monoclonal antibodies against CD3 (1 μg/ml, clone OKT-3, BioXcell) and CD28 (1 μg/ml, clone CD28.2, BioLegend). For the Th17 or Tc17 polarization assays, the following cytokine cocktail was used: human IL-6 (20 ng/ml), TGF-β1 (5 ng/ml), IL-1β (10 ng/ml) and IL-23 (10 ng/ml). For the Th1 polarization assays, cells were incubated with IL-12 (10 ng/ml) and anti-IL-4 antibody, for the Th2 polarizations, cells were cultured with IL-4 (10 ng/ml) and anti-IFN-γ antibody (5 μg/ml). No cytokine cocktail was added for the Th0 polarization assays.

The compound was added at the beginning of the cell cultivation and supernatants were collected after 48 or 72 hrs of incubation. In order to determine whether cpd 1 inhibited IL-17A production from polarized Th17 cell cultures, CD4^+^ T-cells were incubated with the Th17-inducing cytokines for 7 days, washed three times to remove any residual IL-17A, followed by re-stimulation of cells (5 x 10^4^ cells/well) for another 72 hrs with CD3 and CD28-specific antibodies in the presence of the cpd 1. Supernatants were collected and IL-2, IFN-γ, IL-13, IL-17A, IL-17F or IL-22 cytokine concentrations were quantified by ELISA according to the manufacturer`s instructions (eBioscience or BioLegend). For compound-induced cytoxicity assessment, 5 μl of WST-1 reagent (Abnova) was added to each well to the 3-day Th17 cell cultures and incubated for 4 hrs at 37°C. Enzymatic cleavage of the tetrazolium salt WST-1 to formazan by cellular mitochondrial dehydrogenases present in viable cells was measured by an EnVision Multilabel Plate Reader (Perkin Elmer).

### Human γδ T-cell culture

Human γδ T-cells were prepared from PBMCs by immunomagnetic isolation using an isolation kit from Stem Cell, according to the manufacturer´s instructions. Cells were seeded at a density of 1 x 10^5^ cells/well in the presence of the inhibitor or DMSO control and were stimulated with PMA (5 ng/ml) and ionomycin (0.5 μg/ml). Following a 24 hr incubation period, supernatants were removed and analyzed for IL-17A using an IL-17A ELISA kit (eBioscience).

### Th17 cell polarization assay using purified rat CD4^+^ T-cells

CD4^+^ T-cells from male Lewis rats were obtained from splenocytes and used in Th17 differentiation assays as previously described [23]. In brief, cells were incubated with the following rat-specific antibodies: CD8a (clone OX8; BioLegend), CD45RA (clone OX33; BioLegend), CD11b/c (clone OX42; BD Biosciences), CD25 (clone OX39; eBioscience), and erythrocytes (clone OX83; Cedarlane), followed by a washing step. Cells were incubated with anti-IgG coated microbeads (Miltenyi Biotec) and unwanted cells were magnetically depleted using a MACS column system according to the manufacturer`s instructions (Miltenyi Biotec). CD4^+^ T-cells were stimulated with rat-specific anti-CD3 (pre-coated to the plated at 4 μg/ml, clone G4.18) and soluble anti-CD28 antibody (2 μg/ml, clone JJ319, BioXcell) together with recombinant human TGF-β1 (5 ng/ml, BioLegend), mouse IL-6 (20 ng/ml, BD Pharmingen), rat IL-1β (10 ng/ml, R&D Systems), and mouse IL-23 (10 ng/ml, BioLegend) in the presence of anti-rat IFN-γ antibody (5 μg/ml, BioLegend). After 72 hrs of incubation, the supernatants were harvested and IL-17A cytokine concentrations were quantified by ELISA according to the manufacturer`s specifications (eBioscience).

### Human whole blood assay

Heparinized whole blood from healthy volunteers was diluted (20% final concentration) with DMEM high glucose medium containing L-Glutamine, supplemented with 5% FCS, 50 μM Mercaptoethanol, 5% Dextran, 100 U/ml penicillin, and 100 mg/ml streptomycin. Cells were stimulated with Con A (10 μg/ml, Sigma) and 10 ng/ml recombinant human IL-23 (BioLegend) in the presence of the RORγt inhibitor. After 72 hrs of incubation at 37°C, the supernatants were collected for IL-17A or IL-2 cytokine quantification by ELISA according to the manufacturer`s instructions (eBioScience or BioLegend).

### Intracellular staining and flow cytometry

For intracellular cytokine staining, Th17 cells obtained from *in vitro* culture after 72 hrs were incubated for 4 hr at 37°C with 5 ng/ml of PMA (Sigma) and 500 ng/ml of ionomycin (Calbiochem) in the presence of 10 μg/ml Brefeldin A (Sigma). Surface staining was performed for 20 min with FITC labelled CD4 antibody. After surface staining, the cells were washed, fixed and permeabilized in Cytofix/Cytoperm (BD Biosciences) according to the manufacturer`s instructions prior to intracellular staining with PE labelled IL-17A antibody. Data were acquired on a LSR Fortessa (BD Bioscience) and analyzed using FlowJo software (Tree Star).

### Quantitative RT-PCR for RORγt-controlled signature gene expression

Total RNA from human Th17 cells (6 x 10^5^ cells) or RORγt-transduced HUT78 cells (4 x 10^5^ cells) that were stimulated with DMSO or Cpd 1 for 72 or 24 hrs, respectively, was extracted using RNeasy kit including a DNAse I digestion step according to the manufacturer`s protocol (Qiagen). cDNAs were prepared with the High Capacity cDNA Reverse Transcription Kit (Applied Biosystems). Quantitative RT-PCR analysis was performed using a TaqManViiA7 (Applied Biosystems). The expression level of each gene was normalized to β-glucoronidase (*Gus*) expression (431088E) using the ΔΔCt method. The following probes were used for RT-PCR: *RORC* (Hs01076112_m1), *IL17A* (Hs00936345_m1), *IL17F* (Hs00369400_m1), *IL26* (Hs00218189_m1), *IL23R* (Hs00332759_m1), *CCR6* (Hs00218189_m1).

### Chromatin immunoprecipitation (ChIP) assay

Histone 3 modifications at the *IL17A* and *IL23R* promoter regions were detected using a chromatin immunoprecipitation (ChIP) assay kit (Qiagen), as per the manufacturer’s protocol. ChIP antibodies to detect H3 acetylation at Lys-9/14 (H3Ac; Qiagen) or H3K4me3 (Abcam) were used for chromatin immunoprecipitation along with an isotype-specific rabbit control IgG Ab (Qiagen). HUT78 cells expressing RORγt or control vector (1 x 10^7^ cells/condition) were left untreated or were stimulated with PMA plus CD3 mAb for 24 hrs. Cells were incubated with cpd 1 (10 μM final concentration) or with DMSO as vehicle control at the beginning of the stimulation. Samples were prepared for ChIP according to the manufacturer`s specification. In short, cells were cross-linked for 10 min with 1% formaldehyde and nuclear extracts were obtained by sonication. Chromatin immune precipitations were performed with 1.5 μg of anti-H3Ac, anti-H3K4me3 or with the respective isotype control Ab. After washing, elution, and reversion of crosslinking, the DNA was isolated and used for real-time qPCR using the RT^2^ SYBR Green qPCR Master Mix (Qiagen) according to the manufacturer`s instruction. Quantitative RT-PCR analysis was performed using a TaqManViiA7 (Applied Biosystems). The following promoters were used for RT^2^ SYBR Green qPCR (all from Qiagen): *IL17A*-6: binding to -5398 relative to the transcription start site (TSS), RefSeq Acession number: NM_002190.2. *IL23R*-1: -115 relative to TSS, *IL23R*+6: 5693 relative to TSS, RefSeq Acession number: NM_144701.2. The enrichment was normalized to input DNA.

### RORγt DNA oligonucleotide pull-down assays

Biotinylated oligonucleotide probes containing putative 4 x RORE or mutated forms thereof were synthesized (Microsynth, Switzerland) and annealed in order to form a double stranded DNA probe that was used for pull-down studies using nuclear extracts originating from HUT78 cells that express RORγt or empty control vector.

The sequences of the 3’ biotinylated probes used were as follows (the bold nucleotides represent a putative RORγt binding site or the mutated form of the RORE):

RORE sense strand: 5**’**GGTAAGT**AGGTCA**TGGTAAGT**AGGTCA**TGGTAAGT**AGGTCA**TGGTAAGT**AGGTCA**TCGTGAC-3**’** Biotin

RORE antisense strand: 3**’**-CCATTCA**TCCAGT**ACCATTCA**TCCAGT**ACCATTCA**TCCAGT**ACCATTCA**TCCAGT**AGCACTG-5**’**

RORE mutated sense strand: **5’**-GGTAAGT**ACCTCA**TGGTAAGT**ACCTCA**TGGTAAGT**ACCTCA**TGGTAAGT**ACCTCA**TCGTGAC-3’ Biotin

RORE mutated antisense strand: 3’-CCATTCA**TGGAGT**ACCATTCA**TGGGAGT**ACCATTCA**TGGAGT**ACCATTCA**TGGAGT**AGCACTG-5’

HUT78 cells stably transduced with eGFP-RORγt (1 x 10^6^ per sample) were stimulated with PMA (5 ng/ml) and ionomycin (0.5 μg/ml) or left unstimulated for 2 hrs at 37°C. Cells were incubated with cpd 1 (10 μM final concentration) 30 min prior to stimulation. Nuclear lysates were prepared using a nuclear extraction kit according to the manufacturer`s instruction (Affymetrix). The nuclear extracts were pre-cleared with streptavidin-sepharose beads (GE healthcare Amersham) for 1 hr at 4°C. Next, the pre-cleared supernatants were incubated with biotinylated double-stranded RORE or with mutated RORE oligonucleotides (5 nM final concentration) for 1 hr at 4°C, followed by addition of a 50% slurry of streptavidin-sepharose beads for 1 hr. The resin-bound complexes were washed five times with 1 ml buffer consisting of 10 mM HEPES, pH 7.9, 100 mM KCl, 1 mM EDTA, 5 mM MgCl_2_, 1% NP-40 and 10% glycerol with freshly added protease inhibitors (Roche). After the final wash, the complexes were dissolved in SDS-PAGE sample buffer (Sigma), boiled and used for Western blotting. The pull-down samples were immunoblotted with RORγ-specific antibody (clone H-190, Santa Cruz Biotechnology). As a loading control, nuclear extracts containing 0.5 x 10^6^ cell equivalent were blotted with RORγt-specific antibody (clone H-190, Santa Cruz Biotechnology). Protein bands were visualized by enhanced chemiluminescence (Western bright Quantum, Advansta).

### Antigen-induced arthritis (AiA) in rats

Female Lewis rats (190–220 g) were sensitized intradermally on the back at two sites with 500 μg of methylated BSA (Sigma) homogenized with CFA (DIFCO) on d -21 and -14. On d 0, the rats received intra-articular injections containing 50 μl of mBSA (10 mg/ml) in 5% glucose into the right knee (antigen injected knee) and 50 μl of 5% Glucose into the left knee (control knee). Cpd 1 was dosed twice daily per oral gavage at 15 or 45 mg/kg starting just before the antigen challenge until the end of the study. Knee swelling was measured using digital calipers and expressed as a ratio of right versus left (control) knee swelling. Sublingual blood and inguinal draining lymph nodes were obtained 7 days post-challenge and cells were used in *ex vivo* recall assays.

#### *Ex vivo* mBSA recall studies

Draining lymph node cells were isolated and single-cell suspensions were prepared. Cells (4 x 10^5^/well) were re-stimulated with mBSA (200 μg/ml) and incubated for 72 hrs. Heparinized sublingual whole blood was diluted with medium and stimulated with 50 μg/ml of mBSA for 96 hrs. Supernatants were removed and IL-17A cytokine concentrations were determined by ELISA according to the manufacturer`s specifications (eBioscience).

The numbers of IL-17A producing, antigen-specific cells were quantified by ELISPOT as recently described [23]. In brief, inguinal draining lymph node cells (1 × 10^5^/well) from compound or vehicle treated rats used in the AiA study were added to 96-well polyvinylidene difluoride (PVDF) plates (Millipore), pre-coated with anti-IL-17 capture Ab (ELISPOT development module, SEL421, R&D Systems). Cells were stimulated with mBSA (200 µg/ml) at 37°C with CO_2_ for 24 hrs. After washing, the plates were incubated with biotinylated anti-mouse IL-17 detection Ab (ELISPOT development module, SEL421, R&D Systems), followed by incubation with streptavidin-AP concentrate. The spots were developed with an ELISPOT blue color module as the substrate (R&D Systems) and the spots were counted with an ELISPOT reader (AID). The results are presented as number of spots/1 × 10^6^ cells.

### Statistics

The means were compared using both ANOVA followed by Dunnett’s test for multiple comparisons (GraphPad Software Corporation Inc. CA). A *P* value < 0.05 was considered significant.

## Results

### Cpd 1 is a potent and selective RORγt inhibitor

We recently described the discovery of a series of imidazopyridine RORγt inverse agonists by a structure-based hit-to-lead optimization effort [21]. An advanced representative of this series, cpd 1 (Fig 1A), was selected for further investigation of *in vitro* and *in vivo* RORγt-related target biology. The compound was tested in a biochemical TR-FRET assay measuring the compound-induced inhibition of a co-activator peptide binding to the human RORγt-LBD. The inhibitor was potent and displaced RIP140 co-factor binding peptide to the RORγt-LBD in a concentration-dependent manner with an IC_50_ value of 19 nM (Fig 1B).

Thermal stability shift assays were conducted to evaluate the effects of cpd 1 on the stability of the RORγt-LBD in an aggregation-based thermo-denaturation assay. Upon compound binding, the RORγt-LBD denatures at a higher temperature compared to the unliganded, apo- RORγt-LBD, resulting in increased Tagg inflection point values (ΔTagg(inf)). The apo-form of the RORγt-LBD showed a Tagg(inf) of 45.7 +- 0.2 ^o^C, whereas cpd 1 incubation with the RORγt-LBD resulted in an increased Tagg(inf) value of 59.4 +- 0.1 ^o^C (Fig 1C). This indicated, that cpd 1 bound with high affinity to the RORγt-LBD leading to ΔTagg(inf) of 13.7 ^o^C.

**Fig 2:**
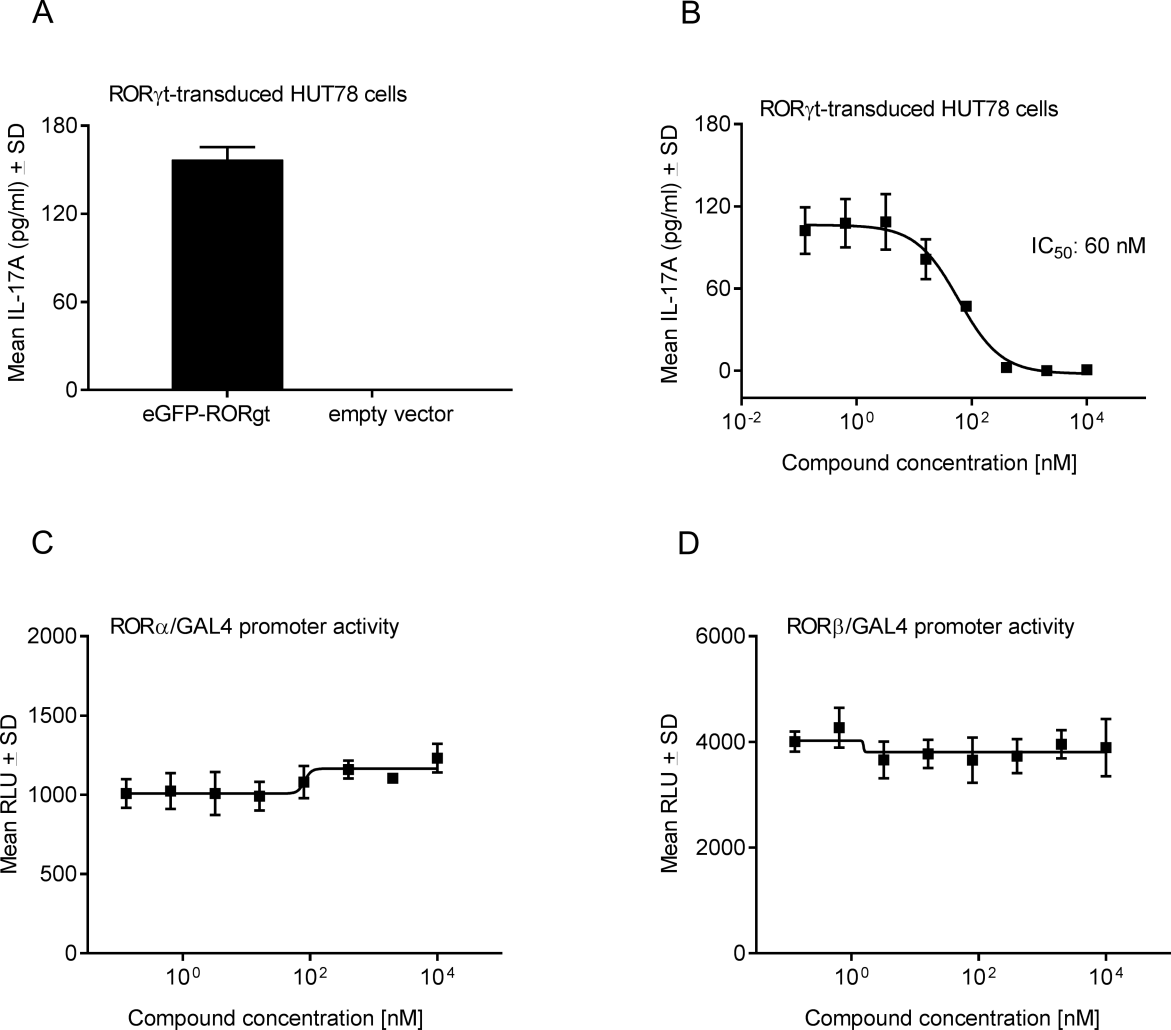
Cpd 1 is a potent and selective RORγt inhibitor in *in vitro* cellular assays. (A) Overexpression of RORγt results in IL-17A production. HUT78 T-cells, stably transduced with human full-length RORγt or control vector, were stimulated with anti-CD3 antibody plus PMA for 48 hrs and IL-17A production was quantified by ELISA. (B) HUT78 T-cells expressing RORγt were incubated with serial dilutions of cpd 1 at the beginning of the stimulation with PMA and anti-CD3 antibody and after 48 hrs IL-17A concentrations were measured by ELISA. Representative example of a concentration-response curve from five independent experiments with triplicate readings is shown. Activity of cpd 1 against the nuclear hormone receptors RORα (C) or RORβ (D). Jurkat T-cells stably expressing GAL4 upstream activator sequences that control the transcription of the luciferase reporter gene were transfected with a GAL4-DNA binding domain fusion construct containing the RORα or RORβ-LBD. Cells were incubated for 24 hrs with cpd 1, followed by cell lysis and measurement of luciferase activity. Graphs are representative of three independent experiments performed in duplicates.

After having demonstrated that binding of cpd 1 to the RORγt-LBD resulted in the displacement of the RIP140 co-activator peptide from the RORγt-LBD in biochemical assays, we next assessed whether cpd 1 showed inhibitory activity towards RORγt in a cellular context. For this, the human T-cell line, HUT78, was transduced with lentiviruses expressing full-length human RORγt and a stable clone was identified that upon stimulation with anti-CD3 Ab plus PMA was a potent IL-17A producer (Fig 2A). Secretion of IL-17A was not detectable after stimulation of HUT78 cells expressing empty control vector, indicating that IL-17A cytokine production was dependent on RORγt expression (Fig 2A). Pharmacological inhibition of RORγt by cpd 1 resulted in complete attenuation of IL-17A secretion in a concentration-dependent manner with an IC_50_ value of 60 nM (Fig 2B).

Subsequently, we addressed the selectivity of cpd 1 towards the closely related nuclear hormone receptor family members RORα and RORβ by performing RORα or RORβ-LBD GAL4 transactivation reporter gene assays. Cpd 1 was devoid of any inhibitory activity towards RORα or RORβ (Fig 2C and 2D), indicating its selectivity for RORγt.

### Cpd 1 selectively suppresses human Th17 and Tc17 cell differentiation

**Fig 3:**
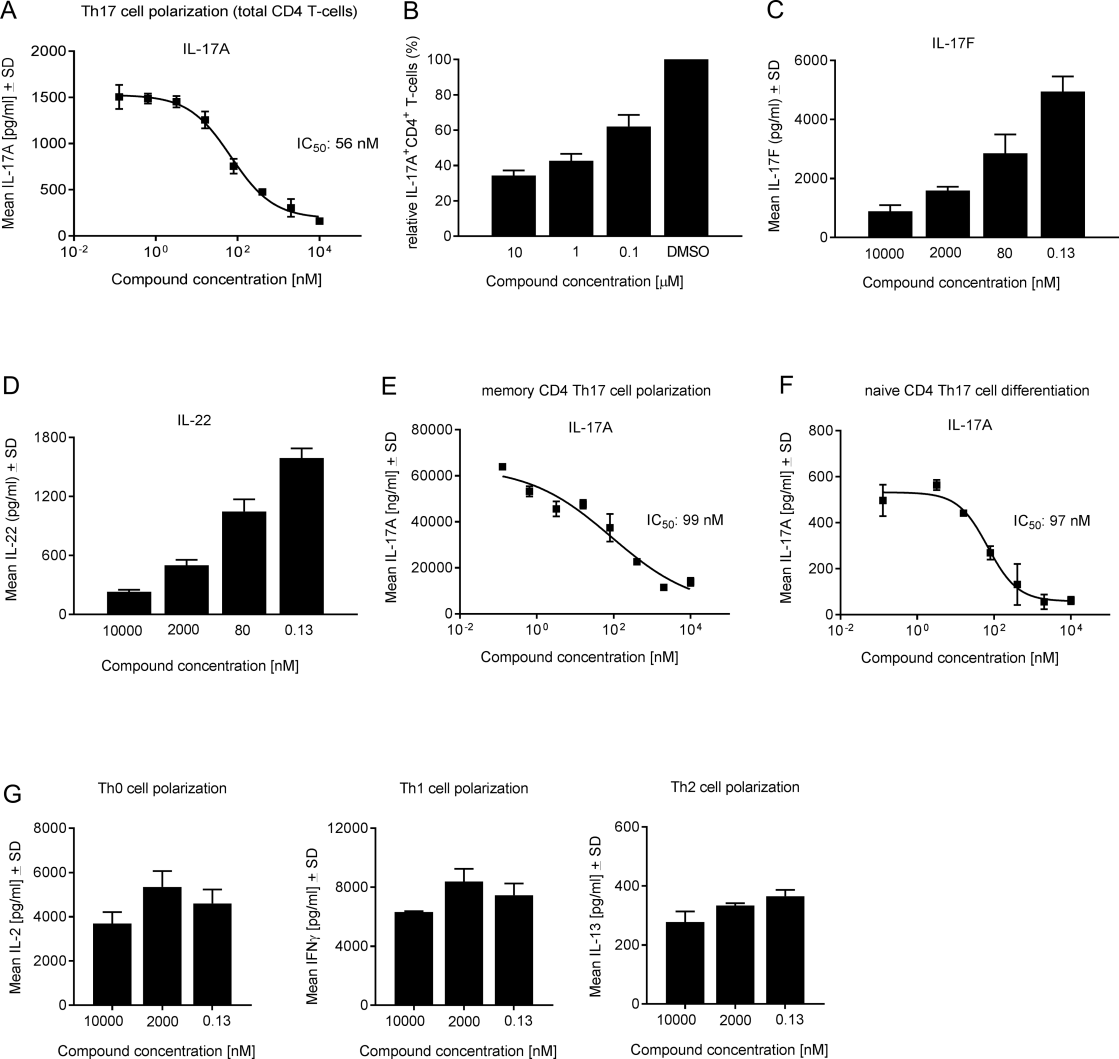
Cpd 1 specifically impairs human Th17 cell polarization and Th17-signature cytokine production. (A) Total CD4^+^ T-cells were incubated with the RORγt inhibitor and were activated under Th17 cell favoring conditions for 72 hrs followed by quantification of IL-17A production. Data are representative of five experiments with triplicate readings. (B) Th17 cells were treated with PMA/ionomycin and Brefeldin A for 4 hrs and frequencies of IL-17A producing cells in gated CD4^+^ T-cells were determined by intracellular staining. Data represent duplicate measurements of two independent experiments. (C and D) Supernatants from polarized Th17 cells were taken to determine IL-17F and IL-22 cytokine concentrations. Naïve (E) and memory (F) human CD4^+^ T-cells were purified from PBMCs and were cultured under Th17 skewing conditions for 7 days in the presence of cpd 1. IL-17A production was quantified by ELISA. Representative examples of concentration-response curves from two experiments with duplicate readings are shown. (G) Human CD4^+^ T-cells were stimulated with anti-CD3 plus anti-CD28 antibodies only (Th0) or incubated with IL-12 and anti-IL-4 antibody (Th1) or with IL-4 and anti-IFN-γ antibody (Th2). After 48 hrs, Th subset signature cytokines including IL-2, IFN-γ and IL-13 were analyzed by ELISAs. Representative examples from two experiments with triplicate readings are shown.

We next focused on studying the inhibitory effect of cpd 1 on Th17 and Tc17 differentiation in primary human T-cells. Total CD4^+^ T-cells were isolated from buffy coats originating from healthy volunteers and cells were stimulated with anti-CD3 plus anti-CD28 specific antibodies together with a Th17 skewing cytokine cocktail. The RORγt inhibitor was added at the start of the Th17 polarization and inhibition of IL-17A production by the compound was assessed after 72 hrs. Cpd 1 was potent in blocking almost completely IL-17A secretion in a concentration-dependent manner with an IC_50_ value of 56 nM (Fig 3A). The impact of the inhibitor on Th17 development was also evident by intracellular IL-17A staining of Th17 cells after 72 hrs of differentiation. The frequencies of IL-17A^+^ cells within the CD4^+^ T-cell population were reduced by up to 65% by the RORγt inhibitor (Fig 3B). Expression of Th17 signature cytokines apart from IL-17A, that were affected by cpd 1 included IL-17F and IL-22 (Fig 3C and 3D). When naïve or memory human CD4^+^ T-cells, were differentiated towards the Th17 cell phenotype, cpd 1 was equipotent at blocking IL-17A production in both CD4^+^ T-cell subsets with IC_50_ values ranging between 71 nM and 96 nM for memory and naïve CD4^+^ T-cells, respectively (Fig 2E and 2F). Cpd 1 acted selectively on the Th17 pathway and it did not interfere with Th0, Th1 or Th2 cell polarizations, hence the production of the signature cytokines (IL-2, IFN-γ and IL-13, respectively) of these Th cell subsets was not affected by the inhibitor (Fig 3G).

To exclude the possibility that cpd 1 mediated these inhibitory effects by inducing cytotoxicity, compound-treated Th17 cells were used in a WST-1 viability assay and no significant effect on cell viability by the RORγt inhibitor was observed (data not shown). These results strongly suggest that the inhibition on the Th17/IL-17 pathway by cpd 1 was not due to impaired cell viability/proliferation, but due to transcriptional inhibition of RORγt.

**Fig 4:**
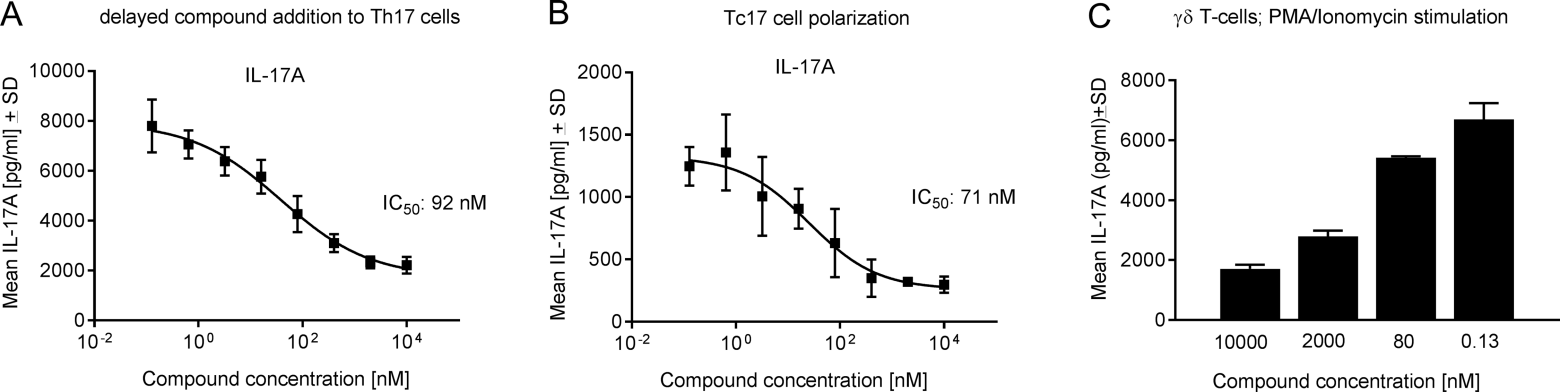
Cpd 1 blocks IL-17A production by differentiated Th17 cells and by Tc17 and γδT-cells. (A) Human CD4^+^ T-cells were stimulated with anti-CD3 plus anti-CD28 antibodies in the presence of Th17 cell promoting conditions for 7 days without compound, followed by extensive washing of cells to remove IL-17A and re-stimulation with anti-CD3 plus anti-CD28 antibodies in the presence of cpd 1. After 3 days, supernatants were collected and IL-17A concentrations were determined. (B) Purified CD8^+^ T-cells were stimulated with anti-CD3 and anti-CD28 antibodies under Th17-polarizing cytokines for 72 hrs, and IL-17A production was quantified. (C) Human γδ T-cells were incubated with cpd 1 and stimulated with PMA/ionomycin for 24 hrs, followed by quantification of IL-17A concentration by ELISA. Representative examples from three independent experiments with triplicate readings are shown.

We further explored whether cpd 1 impaired IL-17A production from already differentiated Th17 cells. To address this, Th17 cells were generated and any IL-17A that was produced during the culture period was removed through washing. Cells were then incubated with the RORγt inhibitor and re-stimulated for a further 72 hrs with CD3 and CD28-specific antibodies in the absence of the Th17-polarizing cytokine cocktail. Delayed compound addition to previously polarized Th17 cells also blocked IL-17A production in a concentration-dependent manner with an IC_50_ value of 92 nM (Fig 4A).

We next investigated the ability of the inhibitor to regulate IL-17A secretion in Tc17 or in γδ T-cells that also express RORγt. Primary human CD8^+^ T-cells were polarized towards an IL-17A producing Tc17 phenotype and cpd 1 impaired IL-17A production in these cells with an IC_50_ value of 71 nM (Fig 4B). The acute, PMA/ionomycin-induced IL-17A production by purified human γδ T-cells was potently inhibited by 74% in a concentration-dependent manner (Fig 4C).

### Downregulation of Th17 cell signature gene expression by cpd 1

**Fig 5:**
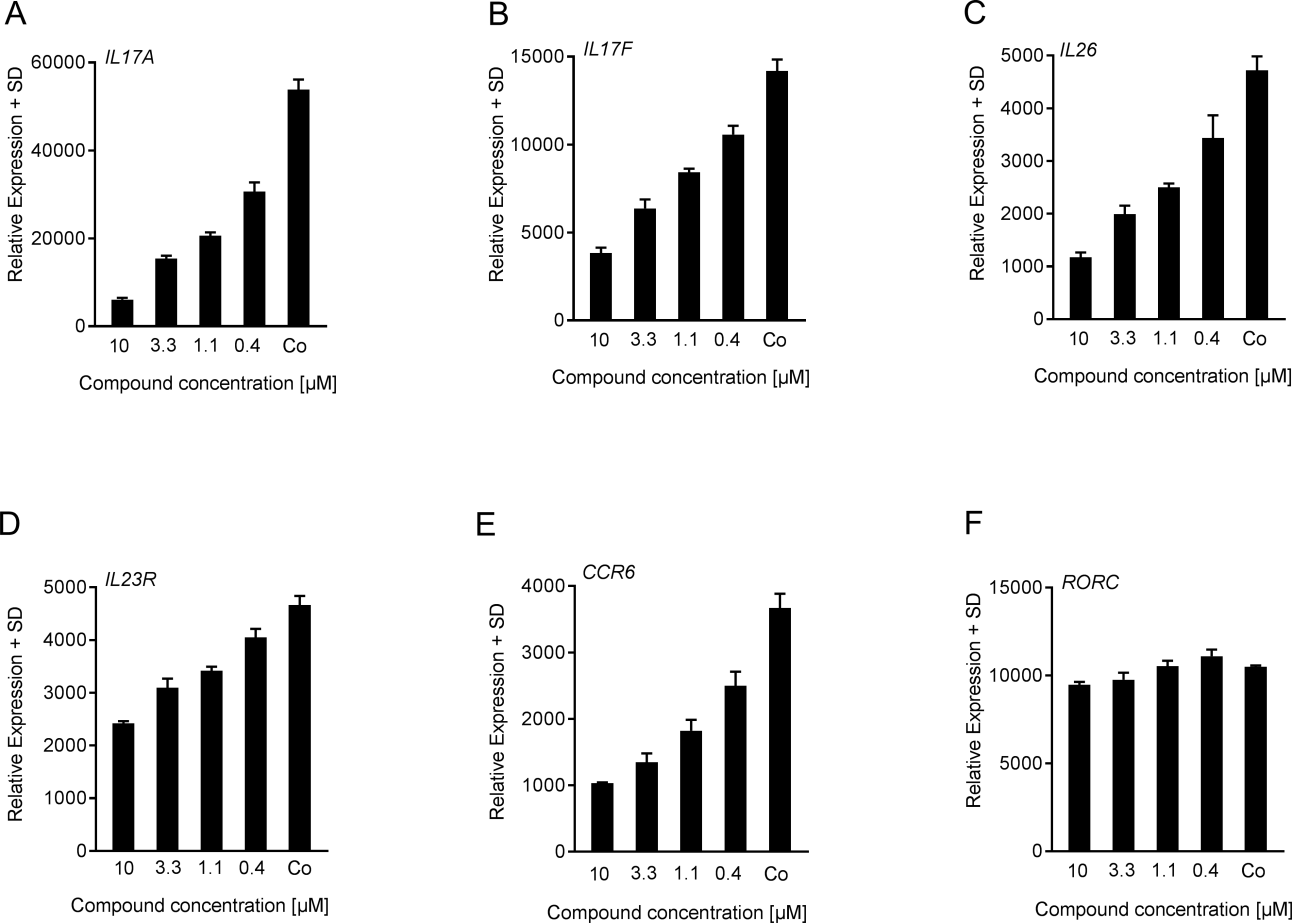
Downregulated RORγt-regulated target gene expression by cpd 1. (A-F) Purified human CD4^+^ T-cells were polarized towards Th17 cells and were treated at the beginning of the cell culture with various concentrations of cpd 1 or with DMSO control (Co). After 72 hrs, mRNA was extracted and transcript levels were quantified by RT-PCR. Gene expression was normalized to β-glucoronidase levels and expressed as arbitrary units. All graphs are representative of three independent experiments containing three technical replicates.

In addition to the cytokines studied above, we next investigated whether gene expression of RORγt-regulated genes [4, 6, 25] was also modulated by cpd 1 in Th17 cells. Human Th17 cells were polarized for 72 hrs in the presence of the RORγt inhibitor followed by quantitative RT-PCR analysis of RORγt-target mRNA levels. Based on the notion that RORγt plays a direct role in controlling expression of various Th17 cell signature cytokines and consistent with the effect on protein expression, we found that cpd 1 reduced Th17 cell-associated mRNA expression including *IL17A*, *IL17F*, *IL26*, *IL23R* and *CCR6* in a concentration-dependent fashion (Fig 5A–5E). The compound had no effect on RORγt gene expression indicating that the inhibitor acted as an inhibitor of the transcriptional activity of RORγt (Fig 5F).

In conclusion, the compound described here consistently and selectively suppressed RORγt-dependent pro-inflammatory gene expression, as well as cytokine production by human primary Th17, Tc17 and γδ T-cells.

### Cpd 1 alters epigenetic regulation at the IL17A and IL23R gene promoters without impairing the DNA binding activity of RORγt

**Fig 6:**
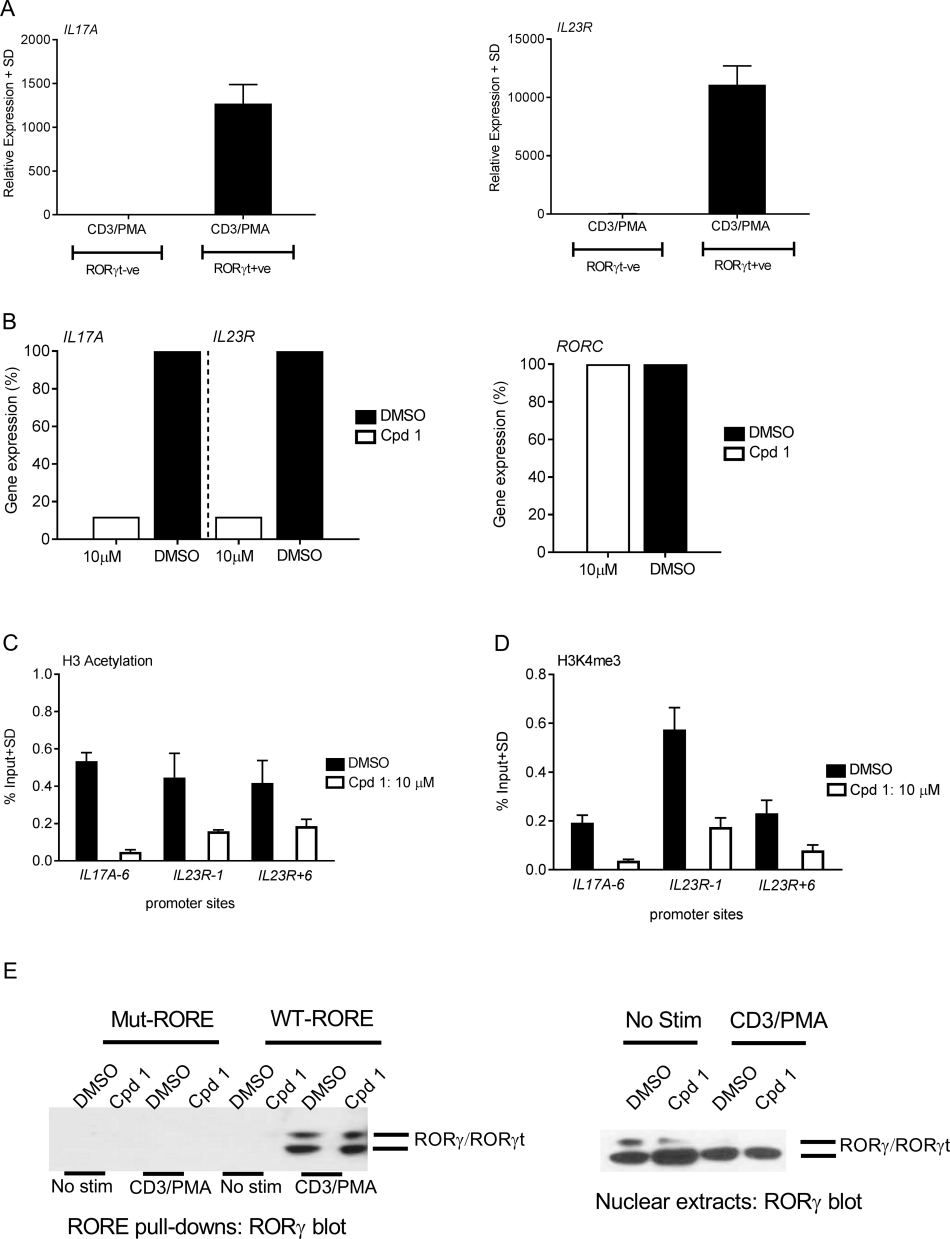
Cpd 1 blocks *IL17A*/*IL23R* gene expression by inhibiting permissive chromatin remodeling at their promoter regions. (A) RORγt or empty vector transduced HUT78 T-cells were stimulated with anti-CD3 antibody and PMA. *IL17A* and *IL23R* gene expression was analyzed by RT-PCR, which was performed on mRNA isolated after 24 hrs of stimulation. (B) RORγt transduced HUT78 T-cells were stimulated as described above in the presence of cpd 1 (10 μM) or DMSO and mRNA was prepared followed by analysis of *IL17A*, *IL23R* and *RORC* gene expression via RT-PCR. Gene expression levels are shown relative to DMSO treated cells (100%). (C and D) Stimulated cell lysates from cpd 1(10 μM) or DMSO-treated RORγt transduced HUT78 T-cells were cross-linked with 1% formaldehyde, sonicated and chromatin preparations were immunoprecipitated with H3K9/14 acetyl or H3K4me3-specific or isotype-specific control antibodies. The precipitated DNA was quantified by qPCR with primers specific for *IL17A* and *IL23R* promoter regions. The results were normalized to an input control. (E) RORγt transduced HUT78 T-cells were treated with cpd 1(10 μM) or DMSO, stimulated with anti-CD3 antibody/PMA or left unstimulated (No stim) for 2 hrs and nuclear extracts were prepared. Nuclear extracts were equally divided into two and each half was subjected to pull-down experiments using mutated or wild-type biotinylated RORE oligonucleotides followed by immobilization of complexes with streptavidin Sepharose beads. After extensive washing pull-down complexes (left panel) or nuclear extracts (right panel) were subjected to SDS PAGE and RORγ Western blot analysis. Graphs and Western blots are representative of two independent experiments.

In order to characterize the mechanism of how cpd 1 suppresses the transcription of RORγt-controlled genes, it was examined whether the inhibitor interfered with DNA accessibility at the genetic loci of *IL17A* or *IL23R* target genes [25–28]. Rather than using Th17 cells, where chromatin remodeling is most likely regulated by various other transcription factors, such as STAT3, IRF4 or BATF, we chose HUT78 cells stably expressing RORγt as a suitable cellular model to examine alterations in epigenetic modifications that occur in a RORγt-dependent fashion. Analysis of *IL17A* and *IL23R* gene expression after PMA and anti-CD3 antibody stimulation of HUT78 cells revealed that these genes were up-regulated only in RORγt expressing cells, whereas these transcripts were undetectable in HUT78 T-cells lacking RORγt (Fig 6A). As expected, *IL17A* and *IL23R* gene expression were markedly suppressed (88%) after treatment with cpd 1 (Fig 6B), confirming that these genes are under the transcriptional control of RORγt. Expression of RORγt mRNA was not altered by the inhibitor (Fig 6B), ruling out that compound-induced differences in target gene expression occur as a result of reduced RORγt expression. The results further reassured us that this cellular system is useful to examine epigenetic alterations occurring as a consequence of RORγt inhibition. After a 24 hr stimulation period with PMA and anti-CD3 antibody, chromatin immunoprecipitation was performed with H3AcK9/K14- or H3K4m3-specific antibodies followed by PCR analysis using primers specific for the *IL17A* or *IL23R* promoters. Permissive histone modifications, such as H3AcK9/K14 and H3K4m3 were evident in DMSO treated cells, whereas addition of cpd 1 prior to the stimulation resulted in a reduced methylation or acetylation state of histone H3 at the *IL17A* or *IL23R* loci (Fig 6C–6D), indicating reduced DNA accessibility induced by the RORγt inhibitor. These findings are clearly in line with the impaired IL-17A cytokine production (Fig 2B) and suppressed *IL17A* and *IL23R* gene transcription observed before (Fig 6B).

The RORγt inhibitor may modulate target gene transcription by mechanisms involving either impaired RORγt occupancy at the target DNA regulatory sites or by interfering with co-activator/co-repressor interactions at the RORγt-LBD while preserving the DNA-binding capacity of RORγt to the RORE sites. We explored whether cpd 1 may affect DNA-binding activity of RORγt by analyzing the occupancy of RORγt to the RORE elements. Nuclear extracts prepared from stimulated RORγt transfectants were subjected to pull-down studies using oligonucleotides containing the consensus binding sequences for this nuclear receptor (AGGTCA) followed by RORγt Western blot analysis. RORγt recruitment to the RORE oligonucleotides was readily detectable in cell lysates expressing RORγt (Fig 6E). By contrast, RORγt binding was undetectable when mutated, non-functional versions of the RORE oligonucleotides were used as a specificity control for the pull-down experiments (Fig 6E). Occupancy of the RORE elements by RORγt was observed in nuclear extracts originating from stimulated cells, but little or no RORγt was bound to the RORE elements in lysates from unstimulated cells. RORγt binding to the RORE elements was still preserved in cells that were treated with 10 μM of cpd 1 and occurred to a similar degree compared with DMSO treated samples (Fig 6E). Similarly, the inhibitor had no impact on RORγt protein expression (Fig 6E), which was consistent with the previous results obtained for RORγt mRNA levels (Fig 6B).

These results suggest that the inhibition of RORγt transcriptional activity by cpd 1 mainly involves blockade of the interaction with co-factors that bind to the RORγt LBD, while leaving the DNA-binding capacity of RORγt intact.

### Cpd 1 attenuates IL-17 production in the presence of human whole blood and blocks rat Th17 polarization

**Fig 7:**
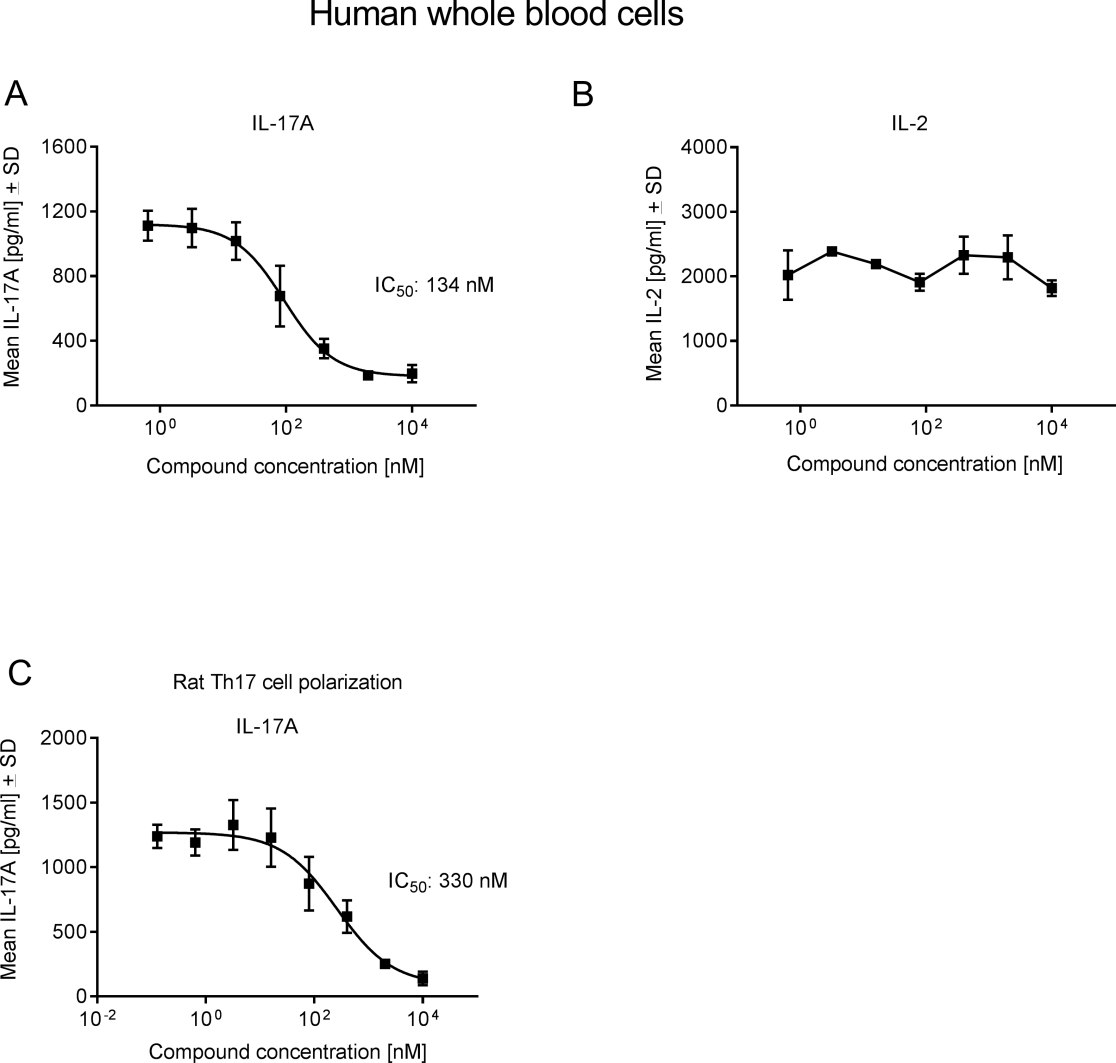
Cpd 1 inhibits IL-17A production by whole blood cells and blocks rat Th17 differentiation. (A and B) Diluted human whole blood cells were stimulated with ConA and IL-23 in the presence of cpd 1. After 72 hrs, IL-17A (A) or IL-2 (B) production was measured by ELSA. Data are representative from five (A) or two (B) independent experiments with triplicate measurements. (C) Purified CD4^+^ Tcells originating from splenocytes from Lewis rats were stimulated with anti-CD3 and anti-CD28 antibodies in the presence of Th17-skewing conditions. After 72 hrs, IL-17A concentrations in supernatants were determined by ELISA. Representative example of concentration-response curve from two experiments with triplicate readings is shown.

It was assessed whether the inhibitor was active in the presence of human plasma and if it was able to inhibit ConA plus IL-23-induced IL-17A production in a human whole blood assay. Cpd 1 was potent and suppressed IL-17A secretion by whole blood cells with an IC_50_ value of 134 nM (Fig 7A), whereas it did not significantly affect the production of the non-RORγt pathway related cytokine IL-2 (Fig 7B). In a Th17 polarization assay using purified rat T-cells, the RORγt inhibitor showed good potency and inhibited IL-17A production with an IC_50_ value of 330 nM (Fig 7C), confirming that the role of RORγt in up-regulating IL-17A production is conserved in human as well as in rat cells. In summary, cpd 1 is a potent and selective inhibitor of RORγt in human and in rat cells *in vitro*.

### Inhibition of RORγt attenuates antigen-induced arthritis (AiA) in the rat

**Fig 8:**
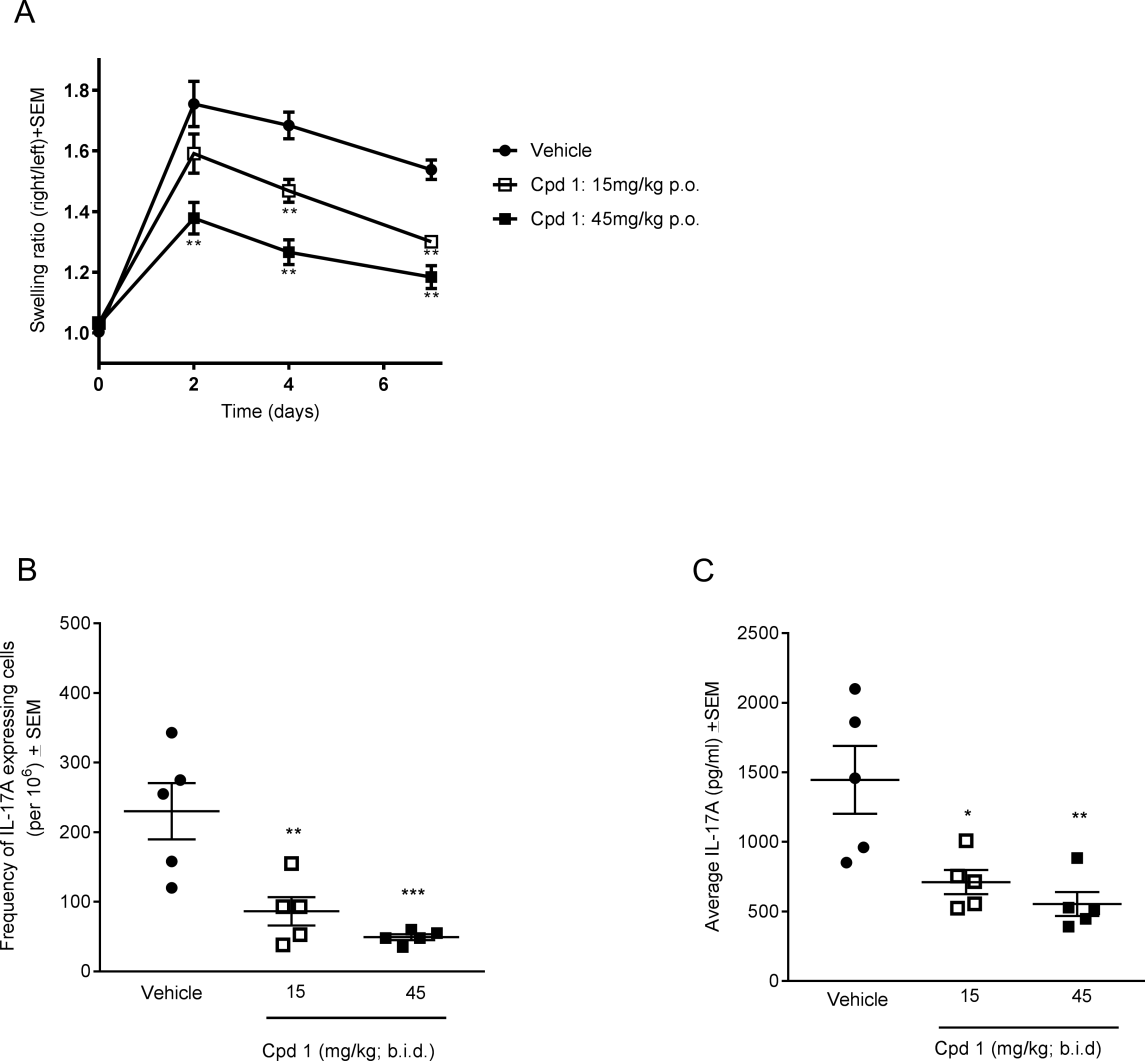
Cpd 1 ameliorates antigen-induced arthritis (AiA) responses in Lewis rats. (A) Rats were immunized twice with methylated BSA (mBSA). Three weeks later, the right knees of the animals were challenged with mBSA in 5% glucose, while the left knees were injected with vehicle (5% glucose). Starting just prior to antigen challenge, cpd 1 was administered twice daily via oral gavage at the indicated doses for 7 days and knee swelling was monitored. The mean ± SEM of the swelling ratios between antigen-challenged and vehicle injected knees are shown (*n* = 5). Results are representative of two experiments with 5 rats per treatment group. (B) Draining lymph node cells were prepared 7 days after mBSA challenge and stimulated *ex vivo* with mBSA (200 μg/ml). Frequencies of IL-17A secreting antigen-specific cells were quantified by ELISPOT after 6 h. (C) Diluted sublingual whole blood cells originating from cpd 1- or vehicle-treated animals were re-stimulated with mBSA (50 μg/ml) and after 96 hrs, IL17A concentrations were determined by ELISA. Each point represents mean ± SEM from an individual rat (n = 4–5 readings/animal). *, P < 0.05; **, P < 0.01; ***, P < 0.001 Dunnett´s test.

Based on the overall promising potency and selectivity profile, further mechanistic *in vivo* studies were conducted with the RORγt inhibitor. We recently published that cpd 1 attenuated IL-17 responses of antigen-specific cells, during a delayed type hypersensitivity reaction in *ex vivo* antigen recall assays [21]. In this report, we investigated the *in vivo* efficacy of cpd 1 in the antigen-induced arthritis (AiA) model in rats. Animals were immunized with methylated BSA (mBSA) in Complete Freund's Adjuvant (CFA) and mono-arthritis was induced two weeks later by a single intra-articular injection of mBSA into the knee joint. Cpd 1 was dosed twice daily per oral gavage at 15 or 45 mg/kg starting immediately prior to antigen challenge until the end of the study (day 7). During the 7-day *in vivo* study, each rat was monitored daily for any potential compound-induced adverse effects. The compound was well tolerated and no obvious signs of discomfort and toxicity during the treatment duration were recorded. Treatment with cpd 1 resulted in a dose-dependent reduction of the knee swelling measurement that was already evident on day 2 post-challenge and persisted until day 7 (Fig 8A). Likewise, the compound was potent and significantly reduced the frequencies of IL-17A producing cells (up to 80% compared to vehicle treated rats) in an *ex vivo* methylated BSA recall assay using inguinal draining lymph node cells (Fig 8B), suggesting that the RORγt inhibitor suppressed the generation of pathogenic T-cells *in vivo*. When whole blood cells were re-stimulated *ex vivo* with methylated BSA, cells from cpd 1 treated rats showed significantly reduced IL-17A responses (up to 62%) compared to vehicle control animals (Fig 8C). The effects of the RORγt inhibitor on antigen-induced IL-17A secretion were dose-dependent. Hence, oral administration of cpd 1 effectively attenuated knee swelling responses in rats and this effect correlated with suppressed IL-17A production.

## Discussion

There is compelling evidence that RORγt represents an attractive drug target based on its critical involvement in the regulation of the Th17/IL-17 pathway that plays a pivotal role in the pathogenesis of several autoimmune disorders, including psoriasis, ankylosing spondylitis, multiple sclerosis, uveitis and rheumatoid arthritis [1–3]. In this article, we report a detailed *in vitro* characterization of an imidazopyridine RORγt inverse agonist recently identified from an extensive chemical optimization campaign [21]. Cpd 1 binds in the ligand binding pocket of the RORγt ligand binding domain as we have recently shown by X-ray crystallography in [29] (where cpd 1 is designated “4”; the coordinates are deposited in the PDB databank (PDB access code = 5M96)). We could show that cpd 1 sterically displaces helix 12 from the agonist position, which leads to decreased interaction between the receptor and the RIP140 co-activator peptide. Thus, the compound acts as an inverse agonist.

Consistent with the role of RORγt in Th17 polarization, cpd 1 impaired expression of pro-inflammatory cytokines, including IL-17A, IL-17F, IL-22 as well as attenuated *IL26*, *IL23R* and *CCR6* gene expression in differentiated human Th17 cells. Because cpd 1 did not affect *RORC* mRNA levels in polarized human Th17 cells (Fig 5E) it cannot be excluded that the inhibitor still allows generation of RORγt expressing Th17 cells to occur. This notion is reinforced by the finding that *IL23R* gene expression is reduced by ca. 50% at the highest compound concentration (Fig 5D) which may still result in potent IL-23 signaling leading to Th17 expansion. However, we unequivocally demonstrated that cpd 1 potently blocked production of Th17-specific effector cytokines. The compound was selective for RORγt and did not affect the transcriptional activity of the closely related nuclear hormone receptors RORα or RORβ. Cpd 1 was equipotent at inhibiting IL-17A production by naïve and memory CD4^+^ T-cells, whereas it did not modulate polarization of other CD4^+^ T-cell linages including Th0, Th1 or Th2 cells. In the clinical setting, therapeutic intervention generally occurs when pro-inflammatory cytokines produced by fully differentiated Th17 effector cells are abundantly expressed. For that reason, it is a requirement for a RORγt low-molecular-weight inhibitor to not only prevent Th17 differentiation from naïve T-cells, but in addition be able to reduce cytokine production from established Th17 effector cells. We isolated human CD4^+^ T-cells and differentiated them towards Th17 cells over a period of 7 days, removed all IL-17A and subsequently added our RORγt inhibitor to investigate if the compound could still inhibit IL-17A production. Cpd 1 was highly potent at suppressing IL-17A cytokine secretion from already established Th17 cells after a 48 hr re-stimulation period (Fig 4A). Therefore, the RORγt inhibitor did not only effectively prevent generation of Th17 cells, but in addition, it blocked pro-inflammatory cytokine production by previously differentiated Th17 effector cells.

In addition to Th17 cells, Tc17 and γδ T-cells are a major source of IL-17A and these cells have been implicated in the pathogenesis of human psoriasis and various mouse skin inflammation models [30–33]. The RORγt inhibitor also effectively suppressed IL-17A secretion produced by Tc17 cells and inhibited acute IL-17A production by γδ T-cells. γδ T-cells constitutively express RORγt and its target IL-23 receptor [34], thus therapeutic targeting of RORγt-dependent responses elicited by these cells may be beneficial in the treatment of autoimmune diseases, such as psoriasis or of the acute stages of multiple sclerosis [35, 36]. In summary, cpd 1 significantly inhibits IL-17A production in differentiating Th17/Tc17 cells as well as in cells with pre-existing RORγt, such as γδ T-cells and memory Th17 cells.

We next investigated the mechanism of functional RORγt inhibition by cpd 1 by analyzing the transcriptional activation-linked histone marks. In particular, we were interested in H3 acetylation and H3 methylation, of the *IL17A* or *IL23R* gene promoters, which are epigenetic events that occur during Th17 differentiation [26, 37]. Nuclear extracts, originating from RORγt transduced HUT78 T-cells, were used in ChIP experiments and demonstrated that *IL17A* and *IL23R* gene expression was selectively up-regulated in these cells (Fig 6A and 6B). Cpd 1 effectively down-regulated the H3 acetylation and H3 trimethylation at the *IL17A* and *IL23R* promoter regions. These compound-induced epigenetic changes directly correlate with the attenuated *IL17A* and *IL23R* gene expression.

Next, we assessed whether the DNA-binding activity of RORγt to RORE DNA sites was affected by the inhibitor, it was found that cpd 1 did not modify binding to the RORE elements. These results suggest that cpd 1-induced repression of chromatin remodeling and target gene expression likely occurs by a mechanism involving blocking co-activator engagement, thereby preventing assembly of transcription factors and/or facilitating co-repressor recruitment to the LBD. A similar type of LBD-dependent mechanism was also reported in studies of other low-molecular-weight inhibitors that did not interfere with the DNA-binding ability to RORγt target gene sequences [15]. Although not shown in this study, co-factors or transcription factors that have been reported to interact with RORγt in various cellular systems could include steroid receptor co-activators [38], p300, the transcriptional co-factor possessing intrinsic acetyltransferase activity [39, 40], hypoxia inducible factor 1α [39] or Runt-related transcription factor [27]. We attempted to identify co-regulators or transcription factors that may be affected by cpd 1, however the results we obtained were inconclusive. Further studies are necessary to identify the relevant co-factors and transcription factors that are critical in this pathway and those which are affected by cpd 1.

While the transcriptional effects of inhibitors with respect to alterations in RORγt target gene occupancy have been studied before [15, 41], this study provides for the first time mechanistic insight into how pharmacological inhibition of RORγt leads to attenuated target gene expression. Here, we identified one mechanism of functional RORγt inhibition by cpd 1 that involved down-regulation of transcriptional-activation linked epigenetic modifications at target promoter sites, such as histone H3 acetylation and methylation.

We continued to examine whether cpd 1 which has adequate physicochemical and pharmacokinetic properties [21] showed efficacy in a mechanistic *in vivo* arthritis model. Oral administration of the RORγt inhibitor to rats resulted in a dose-dependent reduction in joint inflammation in this T-cell dependent model [42], as well as impaired IL-17A production by draining lymph node cells in an antigen-specific recall assay. It has been described before that RORγt inhibitors had beneficial effects in various mouse inflammatory disease models [15–19, 43, 44], we demonstrate here for the first time that an orally available RORγt inhibitor showed good *in vivo* efficacy in a rat antigen-induced arthritis model. The compound may act by suppressing the Th17/IL-17A pathway by blocking the *de novo* differentiation of naïve Th17 cells and/or by blocking acute IL-17A release originating from effector memory T-cells or by γδ T-cells that were shown to be pivotal for the joint destruction in this disease model [45].

Targeting RORγt by a low molecular weight inhibitor may not only attenuate Th17 responses, but could also affect the frequencies of regulatory T-cells (Tregs). Treg and Th17 cells are reciprocally regulated, depending on the balance between the expression of RORγt and FoxP3 and on the cytokine environment [46]. Skewing the Th17/Treg cell ratio towards the Treg pathway as a result of RORγt inhibition could constitute an attractive therapeutic strategy resulting in additional benefit by inducing immune tolerance. This could be experimentally tested e.g. in an ocular-mediated immune tolerance model to type II collagen that was shown to result in impaired DTH responses elicited by type II collagen [47, 48].

On a similar aspect, there is evidence that tolerogenic dendritic cells are generated by GM-CSF which in turn could promote tolerance by enhancing Treg proliferation and by suppressing Th17 cells [49–52]. Preliminary data from our lab suggest that the RORγt inhibitor does not affect GM-CSF production by Th17 cells. It is therefore possible that GM-CSF produced by Th17 cells may act immunosuppressive and expand Tregs and may act in synergy with cpd 1 resulting in alleviation of autoimmunity. However, depending on the cytokine milieu and the disease context, GM-CSF can have inflammatory properties leading to exacerbation of autoimmunity [53, 54]. Further studies are warranted to determine whether GM-CSF synergizes with our RORγt inhibitor in the downregulation of inflammatory diseases. Currently, we have no evidence that RORγt inhibition leads to CD4^+^ Treg expansion because a structurally related analogue of cpd 1 that had a very similar pharmacological profile did not affect Treg number *in vitro* (data not shown). It remains to be established whether our RORγt inhibitor promotes CD8^+^ Treg numbers and whether enhanced Treg differentiation occurs in arthritic rats *in vivo* as was shown by the pentacyclic triterpenoid ursolic acid [55].

Due to the fact that RORγt inhibition attenuates expression of multiple pro-inflammatory cytokines that are implicated in autoimmune diseases, low-molecular-weight inhibitors of this nuclear receptor is expected to be more efficacious in the clinic as compared to existing modalities, which target single cytokines or cytokine receptors of the Th17 pathway.

However, it should be noted that inhibiting Th17-related effector cytokines with a RORγt inhibitor could also result in adverse side effects as these cytokines are crucial for host immunity against infections, such as *Mycobacterium tuberculosis* or *Candida albicans* and may have a protective role in tissue inflammation. Complete attenuation of Th17 signature gene expression was not observed (Fig 5), and as a result RORγt inhibition may not have a detrimental effect on host defense or tissue homeostasis in the clinical setting. Protective functions have also been described for IL-22 [56–59] and cpd 1 impaired IL-22 production in differentiated Th17 cells. However, IL-22 is the hallmark cytokine for Th22 cells that have the aryl hydrocarbon receptor as their master regulator receptor instead of RORγt [60, 61]. It should be noted that there might be a potential safety risk associated with chronic blockade of RORγt function. In particular, thymic alterations and a risk of T-cell lymphoma development may emerge as a consequence of sustained pharmacological inhibition of RORγt as was observed in mice with a genetic deficiency for both RORγ/RORγt isoforms [62, 63]. Similarly, we recently reported the occurrence of thymic aberrations after prolonged treatment of rats with a RORγt inhibitor which were reminiscent to those observed in RORγ/RORγt mice prior to development of thymic T-cell lymphoma [23]. However, pharmacological inhibition of RORγt over a long duration is unlikely to reach 100% and it may be that thymocytes that still express functional RORγt may be able to prevent the occurrence of thymic changes, as was recently described for adult mice that were resistant to lymphoma development and that carried remnant RORγt transcripts due to incomplete disruption of the *RORγ* locus [63]. Clearly, ongoing pre-clinical and clinical studies will be required to address whether thymic T-cell lymphomas observed in RORγ-deficient mice will translate into the clinic. Alternatively, limiting systemic exposure through the development of a topical RORγt inhibitor could be an option for clinical development, this approach is being pursued currently [41].

In summary, we have developed a highly potent selective RORγt inhibitor that showed good efficacy in an *in vivo* mechanistic model and therefore holds considerable potential for the treatment of Th17-dependent diseases, including psoriasis, psoriatic arthritis, and ankylosing spondylitis.

## Supporting information

S1 Table RIP140 co-factor displacement from the ligand binding domain. (XLSX)

S2 Table Cpd 1 is a potent and selective inhibitor in in vitro cellular assays. (XLSX)

S3 Table Cpd 1 specifically impairs human Th17 cell polarization and Th17-signature cytokine production. (XLSX)

S4 Table Blockade of IL-17A production by differentiated Th17 cells, Tc17 and gamma delta T-cells. (XLSX)

S5 Table Downregulated target gene expression by Cpd 1. (XLSX)

S6 Table Inhibition of IL-17A production by whole blood cells and rat Th17 differentiation. (XLSX)

S7 Table Attenuated IL-17 responses in ex vivo recall assays by Cpd 1. (XLSX)
